# Accurate hybrid template–based and MR-based attenuation correction using UTE images for simultaneous PET/MR brain imaging applications

**DOI:** 10.1186/s12880-018-0283-3

**Published:** 2018-11-06

**Authors:** Jakub Baran, Zhaolin Chen, Francesco Sforazzini, Nicholas Ferris, Sharna Jamadar, Ben Schmitt, David Faul, Nadim Jon Shah, Marian Cholewa, Gary F. Egan

**Affiliations:** 1 Monash Biomedical Imaging, Monash University, Melbourne, Australia; 2 Department of Biophysics, Faculty of Mathematics and Natural Sciences, University of Rzeszow, Rzeszow, Poland; 3 Institute of Nuclear Science Polish Academy of Science, Krakow, Poland; 4 Department of Electrical and Computer Systems Engineering, Monash University, Melbourne, Australia; 5 Monash Imaging, Monash Health, Clayton, Australia; 6 Monash Institute of Cognitive and Clinical Neurosciences and School of Psychological Sciences, Monash University, Melbourne, Australia; 7 Australian Research Council Centre of Excellence for Integrative Brain Function, Monash University, Melbourne, Australia; 8 Siemens Healthcare Pty Ltd, Sydney, Australia; 9 Siemens Healthcare Pty Ltd, New York, USA; 10 Institute of Neuroscience and Medicine, Forschungszentrum Juelich GmbH, Juelich, Germany

**Keywords:** PET/MR, Attenuation correction, Brain, UTE

## Abstract

**Background:**

Attenuation correction is one of the most crucial correction factors for accurate PET data quantitation in hybrid PET/MR scanners, and computing accurate attenuation coefficient maps from MR brain acquisitions is challenging. Here, we develop a method for accurate bone and air segmentation using MR ultrashort echo time (UTE) images.

**Methods:**

MR UTE images from simultaneous MR and PET imaging of five healthy volunteers was used to generate a whole head, bone and air template image for inclusion into an improved MR derived attenuation correction map, and applied to PET image data for quantitative analysis. Bone, air and soft tissue were segmented based on Gaussian Mixture Models with probabilistic tissue maps as a priori information. We present results for two approaches for bone attenuation coefficient assignments: one using a constant attenuation correction value; and another using an estimated continuous attenuation value based on a calibration fit. Quantitative comparisons were performed to evaluate the accuracy of the reconstructed PET images, with respect to a reference image reconstructed with manually segmented attenuation maps.

**Results:**

The DICE coefficient analysis for the air and bone regions in the images demonstrated improvements compared to the UTE approach, and other state-of-the-art techniques. The most accurate whole brain and regional brain analyses were obtained using constant bone attenuation coefficient values.

**Conclusions:**

A novel attenuation correction method for PET data reconstruction is proposed. Analyses show improvements in the quantitative accuracy of the reconstructed PET images compared to other state-of-the-art AC methods for simultaneous PET/MR scanners. Further evaluation is needed with radiopharmaceuticals other than FDG, and in larger cohorts of participants.

## Background

Positron Emission Tomography and Magnetic Resonance (PET/MR) scanners allow for simultaneous data acquisition of both modalities and are a very powerful tool for diagnostic and research imaging. Metabolic information obtained from PET scans, combined with the excellent anatomical and functional contrast derived from MRI examinations, provides new possibilities in medical imaging research. In the oncological applications [1–3], where excellent MRI soft tissue contrast distinguishes between soft tissues better than Computed Tomography (CT). PET/MR has also shown promising results in application to neurological studies [4–8]. An additional advantage of PET/MR imaging, compared to PET/CT, is the absence of radiation exposure from CT examination. However, there are a number of important limitations for simultaneous PET/MR imaging, which need further work including accurate attenuation correction of the PET data [9]. This factor is crucial for quantitative PET data analysis in PET/MR scanners, and still remains an active area of methodological development.

Due to the MRI signal properties, the extraction of attenuation coefficients for 511 keV photons detected using PET is not as straightforward as it is in the case of CT [10–12]. The MRI signal depends on many factors, particularly on the proton distribution and relaxation time of the tissue, whereas the CT signal is electron-density and X-ray spectrum dependant. For the most commonly used sequences in MRI, the signal from cortical bone has a similar intensity as that from air, due to the very short T2* relaxation time. Accurate attenuation factor maps are crucial for precise PET data reconstruction, particularly for neurological imaging applications. Significant image artifacts and spatial biases in PET images are found in brain tissue adjacent to the cortical bone, which absorb positron annihilation photons much more strongly than soft tissues. Difficulties in separating cortical bone from air (and/or ignoring bone) when the attenuation correction is based on MR data also contribute to the artifacts [13, 14]. All of these factors necessitate the development of an improved technique for MR-based attenuation correction, especially for neurological applications where demands on the accuracy of tracer quantitation are high.

Currently the two commercially available simultaneous PET/MR scanners have a number of attenuation correction techniques. The Biograph mMR (Siemens Healthcare GmbH, Erlangen, Germany) provides three different techniques, including a method that employs two-point Dixon water and fat imaging and image segmentation [15]. This sequence is not recommended for brain applications, due to an underestimation of PET uptake results [16]. A second technique segments images into bone, soft tissue and air using a dual ultrashort echo time sequence (dUTE) to capture the signal from bone, whilst a third atlas-based technique produces attenuation correction maps using only Dixon images [17, 18]. The Signa PET/MRI (GE Healthcare, Waukesha, WI, USA) offers a 4-compartment class whole-body atlas-based technique [19].

There are three main groups of attenuation correction methods: PET based; MR segmentation based; and MR-CT atlas/template based. The main concept behind PET based methods is to optimise the reconstruction based methods to reconstruct attenuation and activity simultaneously (MLAA) [20]. Extensions of this method have been developed for both time-of-flight (TOF) [21, 22] and non-TOF [23] applications. Due to the high computational demands MLAA based techniques are not practical for clinical applications. One of the most interesting approaches in recent years utilises the background radiation of PET scintillators for the simultaneous acquisition of transmission and emission data [24].

Segmentation-based methods generate attenuation correction maps by relying only on MR images. The attenuation maps are typically segmented into several tissue types and specific attenuation coefficient values are assigned to them. The most commonly used images for tissue segmentation are UTE images [25–29]. In order to assign the continuous attenuation coefficients values for bone, R2* map approaches are typically used, calculated as follows [26, 29]: $R{2}^{\ast }=\frac{\mathit{\log}{I}_1-\mathit{\log}{I}_2}{TE_2-{TE}_1}$, where *I*_1_ and *I*_2_ are the first and second echo images and *TE*_1_, *TE*_2_are the echo times. Moreover, due to rapidly growing computational capabilities, increasingly sophisticated methods such as machine learning and deep learning are being employed for tissue segmentation [30–33]. A number of methods also use T1 images [34] or improved/modified UTE sequences [35–38].

The MR-CT atlas/template-based methods produce pseudo-CT images based on CT-MR databases. Subject images are typically co-registered to the atlas or template, which allows for the assignment of continuous attenuation coefficient values for all voxels, but does not take into account subject-specific variability. Typically, T1w/T2w images are used for this purpose [39–44], with UTE images used rarely [45, 46]. There are also methods that combine atlas based approaches with machine learning or probabilistic methods [47–49]. A more detailed description of the existing attenuation correction methods can be found in [50, 51].

The aforementioned attenuation correction techniques in the literature are usually compared with the vendor’s techniques and a reference CT-based attenuation map. Detailed comparisons between attenuation correction techniques developed by academic groups and by one vendor have been undertaken by [52, 53]. Cabello and co-workers performed an assessment of four well established methods and the Siemens’s UTE method in comparison to a reference CT-based attenuation correction map using data from a cohort of 15 patients who have been administered ^18^F-FDG. A more comprehensive comparison was undertaken in the [53] paper, including evaluation of eleven independent techniques. Three different cohorts from two centres with the overall number of 359 patients were retrospectively analysed. The best overall performance was achieved with atlas-based and segmentation-based techniques. Despite the large number of techniques that were included in the study and the number of patients that were examined, the authors conclusions require further investigation due to the use of CT images as a reference and the necessity to investigate the techniques in younger patients and in patients with focal lesions.

In this work, we introduce a new segmentation technique segmented UTE (sUTE) to generate improved attenuation correction maps for neurological applications. A UTE template based on manual segmentation of the head image is used to segment air, bone and tissue. Two different methods are used to generate attenuation correction maps. The first method assigns fixed attenuation coefficients within a tissue type, and the second method employs subject-specific information from the R2* map to estimate continuous attenuation coefficients for bone. Whilst these methods appear to be similar to other published techniques [26, 41], there are substantial differences between the published techniques and the sUTE methods. The segmentation step with sUTE is fully based on the first echo UTE images, whereas the RESOLUTE technique [26] relies on dual echo UTE images and does not employ Gaussian Mixture Models. The technique developed by Anazodo and co-workers [41] utilises Dixon-based attenuation maps for soft tissue and air, and segments the T1 weighted image to extract a bone mask. The segmentation also involves the Gaussian Mixture Models, albeit tissue probabilistic maps covering only the standard MNI (Montreal Neurological Institute) template space, and does not cover the whole head as in the sUTE method. In this study, we constructed attenuation correction maps based on manually segmented MR images as a reference and investigated the differences between CT-based attenuation correction maps and our manual segmentation based attenuation maps. We also studied the impact of different attenuation coefficient assignments for white matter (WM), grey matter (GM) and cerebrospinal fluid (CSF) on the intensity values in reconstructed PET images. The results from the proposed methods are compared to the vendor UTE-based method and other state-of-the-art methods.

## Methods

PET-MRI datasets were acquired at Monash Biomedical Imaging, Monash University, Melbourne, Australia, between July 2016 and November 2016. All scans were performed using a fully-integrated PET/MR scanner (Siemens Biograph mMR, Siemens Healthcare, Erlangen, Germany with the software version VB20P). All examinations were approved by the Monash University Human Research Ethics Committee.

CT-MRI datasets were acquired at Institute of Neuroscience and Medicine, Forschungszentrum Juelich GmbH, Juelich, Germany and PET/CT Radiologie/Nuklearmedizine UKD Dusseldorf. All MRI scans were performed with a 3 T MRI scanner (MAGNETOM Trio Tim, Siemens Healthcare, Erlangen, Germany with the software version VB13A) equipped with Siemens BrainPET inserts. All CT scans were acquired with two scanners: Philips GEMINI GXL16 PET/CT and Siemens Biograph 128 PET/CT.

### Healthy subjects

The group of subjects examined using PET consisted of five healthy participants with [^18^F]-FDG intravenously administrated at a constant infusion rate of 36 ml/hour for 95 min. Additionally, one healthy female participant was scanned with the same MRI protocol, but without administration of the PET radiopharmaceutical. The MRI images of this participant were used to create the UTE1 (UTE first echo image) template used for bone and air segmentation. The group of five participants who undertook the PET examination are referred to as the PET group throughout this paper.

The group of subjects examined using CT and MRI consisted of four (76.8 ± 6.7 years) healthy participants who undertook PET/CT and PET/MR acquisition. This group is referred to as the CT group throughout this paper.

### Imaging protocol

#### PET imaging protocol in the PET group

Information about the participants in the PET group is given in Table 1. 95-min PET data acquisition started at the same time as radiotracer administration. The PET data were acquired in the list mode, and the one frame 95-min data reconstruction was performed offline using e7tools software provided by Siemens. Ordinary Poisson - Ordered Subset Expectation Maximization (OP-OSEM) algorithm with Point Spread Function (PSF) correction was used with 3 iterations, 21 subsets and 344x344x127 (voxels size: 2.09 × 2.09 × 2.03 mm^3^) reconstruction matrix size. A 5-mm Gaussian post-filtering was applied to the final reconstructed images. The PET protocol was the same for all studies.

**Table 1:** PET group information^a^

Radiotracer	Age average (SD) in years	Female/male	Injected radiotracer (SD) activity in MBq
[^18^F]-FDG	33(9.9)	4/1	82(29)

^a^Table does not include one additional participant who underwent MRI scan without radiotracer administration

#### MRI imaging protocol

Three sequences were acquired simultaneously with the PET acquisition for the PET group to use for evaluating the attenuation correction methods. The dUTE-AC sequence was acquired with the following parameters: echo time 1 (TE1)/echo time 2 (TE2)/repetition time (TR) = 0.07/2.46/11.94 ms, flip angle 10°, voxel size: 1.56 × 1.56 × 1.56 mm^3^, matrix size: 192x192x192. The dual-echo Dixon sequence was acquired with TE1/TE2/TR = 1.23/2.46/3.6 ms, flip angle: 10°, voxel size: 2.08 × 2.08 × 2.34 mm^3^, matrix size: 192x126x128. Lastly, a T1w MPRAGE was acquired with TE/TR = 2.34/1640 ms, flip angle: 8°, voxel size: 1x1x1 mm^3^, matrix size: 256x256x176. The overall MR acquisition time was approximately 9 min.

The dUTE sequence was acquired with the following parameters: TE1/TE2/TR = 0.07/2.46/200 ms, flip angle 15°,voxel size: 1.67 × 1.67 × 1.67 mm^3^, matrix size: 192x192x192 for the subjects included within the CT group.

#### CT imaging protocol in the CT group

The protocol parameters for each subject in CT group are presented in Table 2.

**Table 2:** CT acquisition setups in the CT group

Participant no.	Scanner	X-Ray Tube Voltage [kVp]	X-Ray Tube Current[mA]	Matrix size	Voxel size [mm^3^]
01	Phillips GEMINI GXL16	120	57	512 × 512 × 320	0.488 × 0.488 × 0.750
02	Siemens Biograph 128	120	55	512 × 512 × 229	0.482 × 0.482 × 1.000
03	Siemens Biograph 128	120	60	512 × 512 × 321	0.482 × 0.482 × 0.600
04	Siemens Biograph 128	120	60	512 × 512 × 380	0.494 × 0.494 × 0.600

### UTE1 template and probabilistic maps creation

The whole head template creation process required several steps. Ground truth air, soft tissue, and bone masks were obtained by manual segmentation of both echo UTE images under the supervision of a clinical radiologist (NF). Images of five participants (four from the PET group, and one with only MRI data) were used. The UTE template (UTE_template_) was created using the first echo UTE images, by applying the combination of affine and non-linear registration transformations (ANTS, PICSL, Philadelphia, PA). The UTE_template_ space was in the subject space. All manually segmented masks were subsequently transformed to the template space by applying corresponding transformation matrices; trilinear interpolation was used. The tissue probability maps (TPM) were then averaged within tissue types to create temporary TPM. Due to the constraint that the sum of all tissue probabilities for a single voxel must be equal to unity, all voxels were normalized. Final air (TPM_air_) and bone (TPM_bone_) tissue probability maps were obtained.

**Fig. 1:**
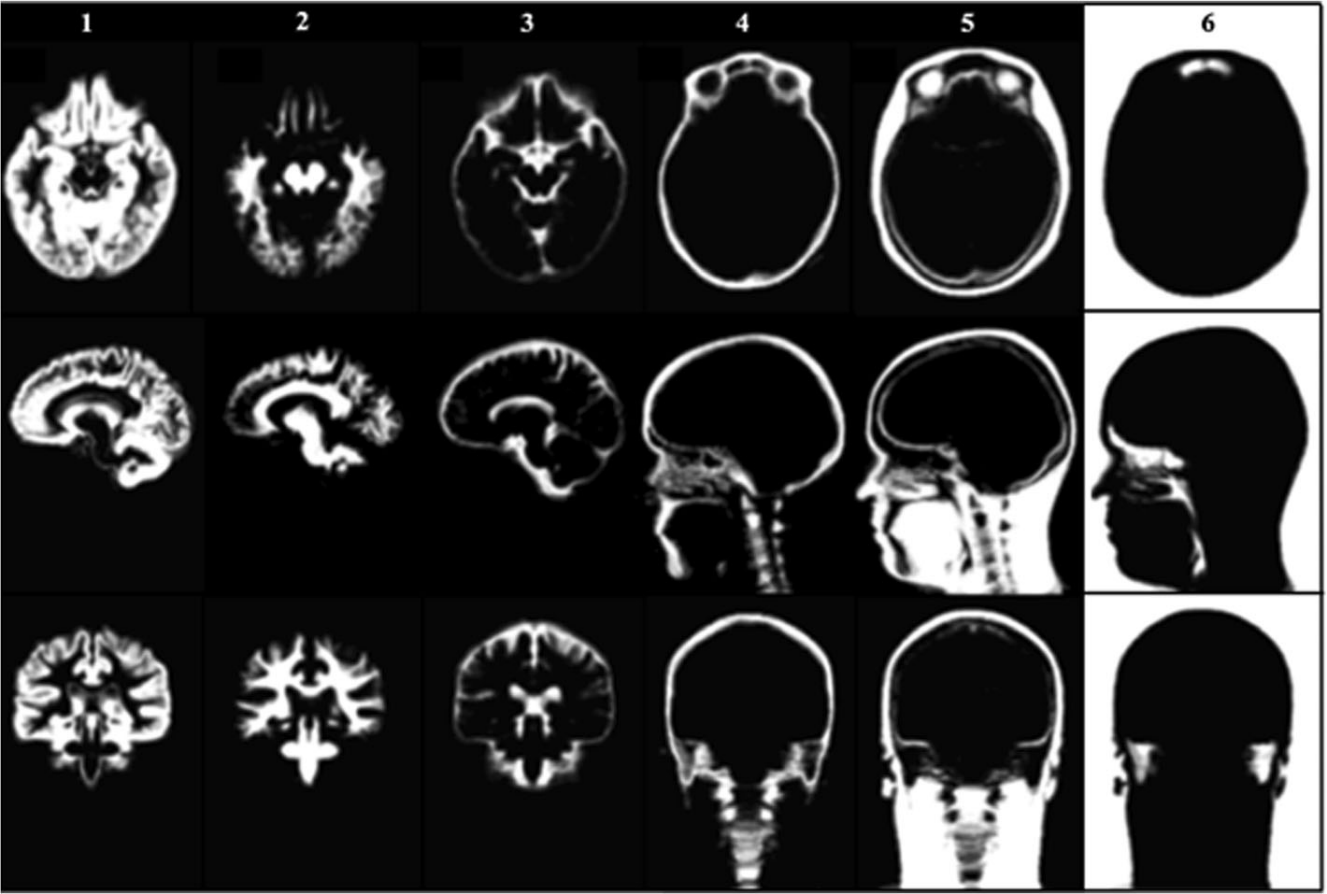
Tissue probabilistic maps used in the study. Tissue probabilistic maps for grey matter (column 1), white matter (column 2), cerebrospinal fluid (column 3), bone (column 4), soft tissue (column 5) and air (column 6) in axial (top), sagittal (centre) and coronal (bottom) view

To generate GM, WM, CSF and soft tissue probabilistic maps, the SPM12 toolbox (Statistical Parametric Mapping, Wellcome Trust Centre for Neuroimaging) [54] was used. Segmentation of the UTE1 images was processed using probabilistic tissue templates provided in SPM12. Bias normalisation was performed prior to segmentation. Using the corresponding transformations, grey matter, white matter, cerebrospinal fluid and soft tissue probability, maps were warped into the template space, averaged and normalised with respect to the air and bone probability maps to obtain final GM, WM, CSF and soft tissue probability maps. If the sum of all TPM within the voxel was greater than 1, the values for air and bone were not changed, but the remaining tissues were rescaled correspondingly. The tissue probability maps for CSF covered only MNI space, and did not include CSF in the spinal region below that space. The tissue probability maps are shown in Fig. 1.

### Attenuation correction map generation

The first echo UTE volume was non-rigidly registered to the UTE_template_ using FSL software [55] with default trilinear interpolation and Correlation Ratio cost function. The SPM12 software was then used to perform the segmentation (Gaussian Mixture Model) using UTE_template_ tissue probability maps as a priori information.

All voxels in the resulting air probability map above 0.1 were identified as air and constant attenuation correction factors equal to 0 cm^− 1^ were assigned. Resulting bone probability maps had a threshold of 0.2 and all values above that threshold were identified as bones. Bone attenuation factors were calculated in two ways: (i) a third order polynomial transformation [26] was used to map R2* intensity vales to attenuation coefficients (AC_sUTEcont_); and (ii) a constant value of 0.151 cm^− 1^ was assigned (AC_sUTEfix_). The attenuation maps were then smoothed with a 2-mm kernel Gaussian filter.

**Fig. 2:**
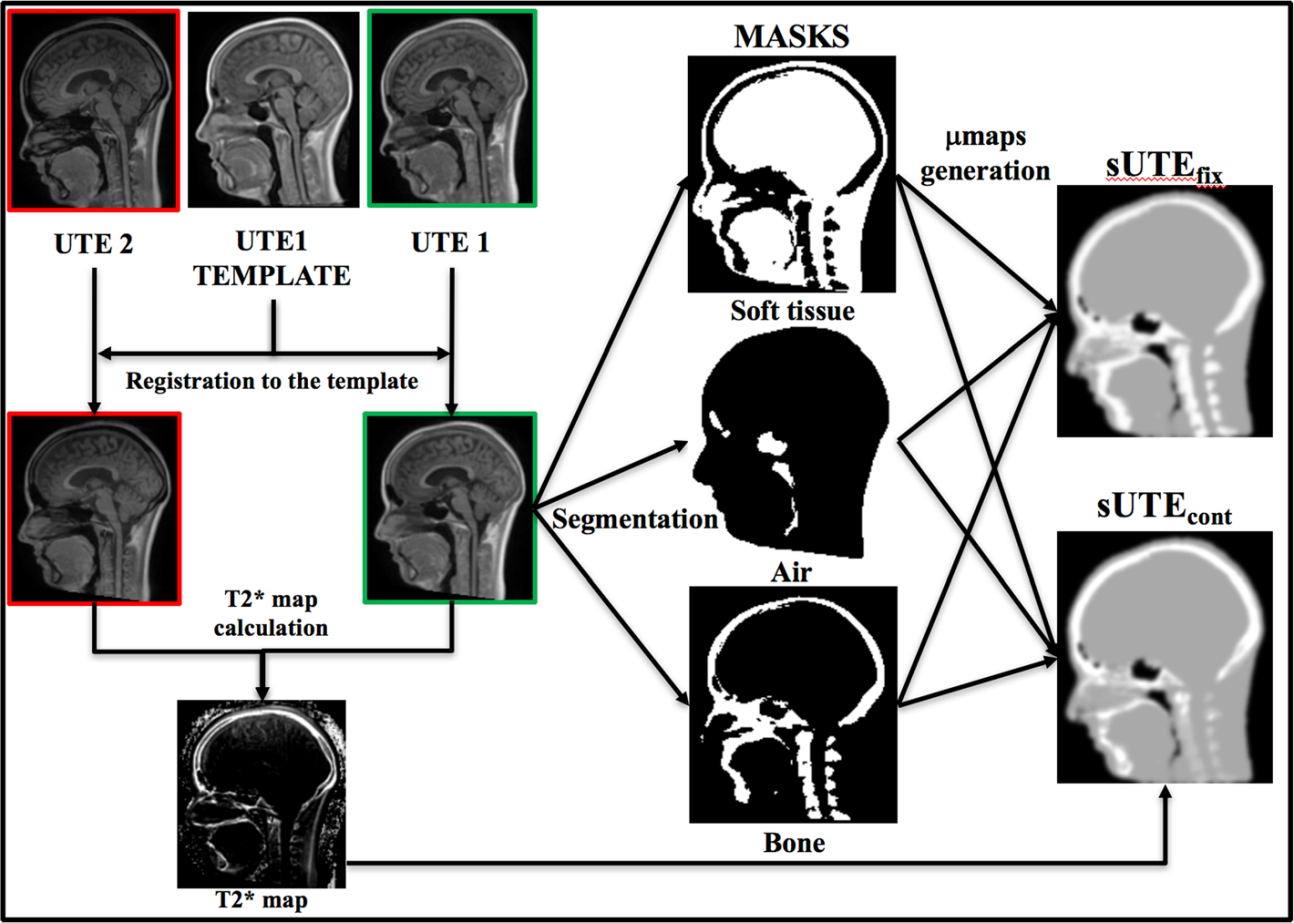
Attenuation maps generation workflow. Both UTE images are rigidly registered to the UTE_template_. Segmentation into three tissue classes (soft tissue, air, bone) is performed using first echo UTE image registered to the UTE_template_. sUTE maps are then generated by assigning attenuation coefficients to all classes with respect to the masks, 3D 2-mm Gaussian smoothing and warping back to the subject space. Attenuation coefficients are fixed for soft tissue and air (0.1 cm^− 1^ and 0 cm^− 1^, respectively), whereas for bones can be either fixed – equal 0.151 cm^− 1^(sUTE_fix_) or continuous – based on R2* map (sUTE_cont_)

For the remaining soft tissue voxels, a 0.100 cm^− 1^ value was assigned. If the template field-of-view (FOV) was smaller than the participant FOV (the template covers only the part of the neck), the vendor provided UTE-based attenuation map values that were used to fill the remaining FOV. The workflow of the attenuation maps generation is depicted in Fig. 2.

Four AC maps were computed and compared as follows:

1. The manually segmented reference attenuation maps had the following attenuation coefficient values assigned for bone, soft tissue, and air respectively: 0.151 cm^− 1^, 0.1 cm^− 1^ and 0 cm^− 1^ (AC_ref_).
2. CT-based attenuation maps were computed by converting HU (Hounsfield Units) maps to the 511 keV attenuation coefficient maps (AC_CT_) [11].
3. The pseudo-CT method proposed by Burgos et al. [39] (AC_UCL_) was based on non-rigid co-registration and similarity measurements between each participant and 41 subjects from a T1w-CT image database. The CT images were generated using the open source software described in [56], converted to attenuation coefficients and smoothed with a 2-mm Gaussian filter. The T1 weighted images were used as inputs. The mean attenuation coefficients were 0.161 cm^− 1^ and 0.096 cm^− 1^ for bone and soft tissue, respectively.
4. The UTE-based (vendor-provided) technique was also evaluated (AC_UTE_).

### Methods for comparing different attenuation correction maps

#### Attenuation correction maps comparison

The segmentation accuracy of AC_UTE_, AC_UCL_ and the proposed method was evaluated using the DICE coefficient, false-positive (FP) and false-negative (FN) rates with respect to the manual segmentation. In case of AC_UCL_, voxels lower than − 500 HU were classified as air, and voxels higher than 300 HU were classified as bone [29]. The classification for AC_UTE_ was performed by setting all voxels with a value equal to 0.151 cm^− 1^ as bone, whereas voxels with a value of 0 cm^− 1^ were assigned as air. A quantitative analysis was conducted for the head region (covering brain and nasal cavities). The DICE coefficient was determined as follows:

1 $$ Dice\ coefficient=\frac{2\bullet \left({AC}_x\cap {AC}_{ref}\right)}{AC_X\cup {AC}_{ref}} $$

where *AC*_*x*_ is a given attenuation map. The mean and standard deviation values across all participants were calculated.

Additionally, for the comparison between AC_CT_ and AC_ref_, difference (*Diff*) and absolute difference (*AbsDiff*) maps were calculated for all subjects. The MNI 2 mm head template was non-rigidly registered to the subject space. The above coefficients were calculated within the head to eliminate the impact from the background.

#### PET reconstructed images comparison

To evaluate PET reconstructed images with respect to PET_ref_ (PET images reconstructed with AC_ref_ attenuation maps), voxel-wise %-difference maps were calculated (Eq. 2.).

2 $$ R=100\bullet \frac{PET_{ref}-{PET}_X}{PET_{ref}} $$

where *PET*_*X*_ stands for PET images reconstructed with one of the evaluated attenuation coefficients maps.

#### Analysis of the test participant

In order to determine the accuracy of bone and air segmentation using the proposed method, the participant who undertook the PET examination, but had not been used to generate the template (referred to as the Test Participant), was also manually segmented into three tissue types: bone, soft tissue, and air. Segmentation was performed under the supervision of the clinical radiologist (NF). The quantitative accuracy of the reconstructed PET images for the Test Participant was assessed, and the normalised relative error across the whole brain was calculated.

#### Whole brain PET performance

To evaluate global PET performance whole brain masks for all participants were created. The 2-mm MNI atlas was aligned to each participant’s PET image using linear and non-linear registration with T1w-MPRAGE images. Inverse transformed brain masks were used to calculate voxel-wise %-difference with respect to PET_ref_ for all participants, defined in *Eq*. 2.

Additionally, the most representative histograms for the brain region were in order to visualise the trend of the differences between the techniques. For all subjects and reconstructed images, we produced histograms with the fixed number of bins (150). The bin width was fixed among methods but varied across subjects due to different uptake level as PET images were not intensity normalized. The 5 × 150 (no. of subjects x no. of bins) matrix were then decomposed using SVD (Singular Value Decomposition) and the most representative histograms were calculated.

#### Regional PET performance

Fifteen predefined regions from the MNI space were inversely transformed to the subjects’ spaces, and the relative error for each participant was calculated. For group analysis, the mean relative error across all participants was calculated. The average and standard deviation volumes of the *RE* (Relative Error) were also calculated. Additionally, Mann-Whitney *U*-tests were performed between the different AC methods. A *p* value of lower than 0.05 was considered statistically significant. Statistical comparisons were not investigated between the PET_sUTEfix_ and PET_sUTEcont_ methods.

#### Effect of AC values for brain soft tissues

To investigate the impact of the different attenuation coefficient assignments for WM, GM and CSF, one additional attenuation coefficient map was produced for the PET group. The following values: 0.151 cm^− 1^, 0.1 cm^− 1^, 0.099 cm^− 1^, 0.099 cm^− 1^, 0.096 cm^− 1^ and 0 cm^− 1^ were assigned for bone, soft non-brain tissue, GM, WM, CSF and air, respectively. The resulting AC maps were smoothed with a 2-mm kernel Gaussian filter (AC_mcAC_).

As in the previous studies, the voxel-wise %-difference between PET_ref_ and PET_mcAC_ were computed with the PET_ref_ used as the reference, and regional %-differences were also investigated.

## Results

### Comparisons of classification accuracy in air and bone in CT group

**Fig. 3:**
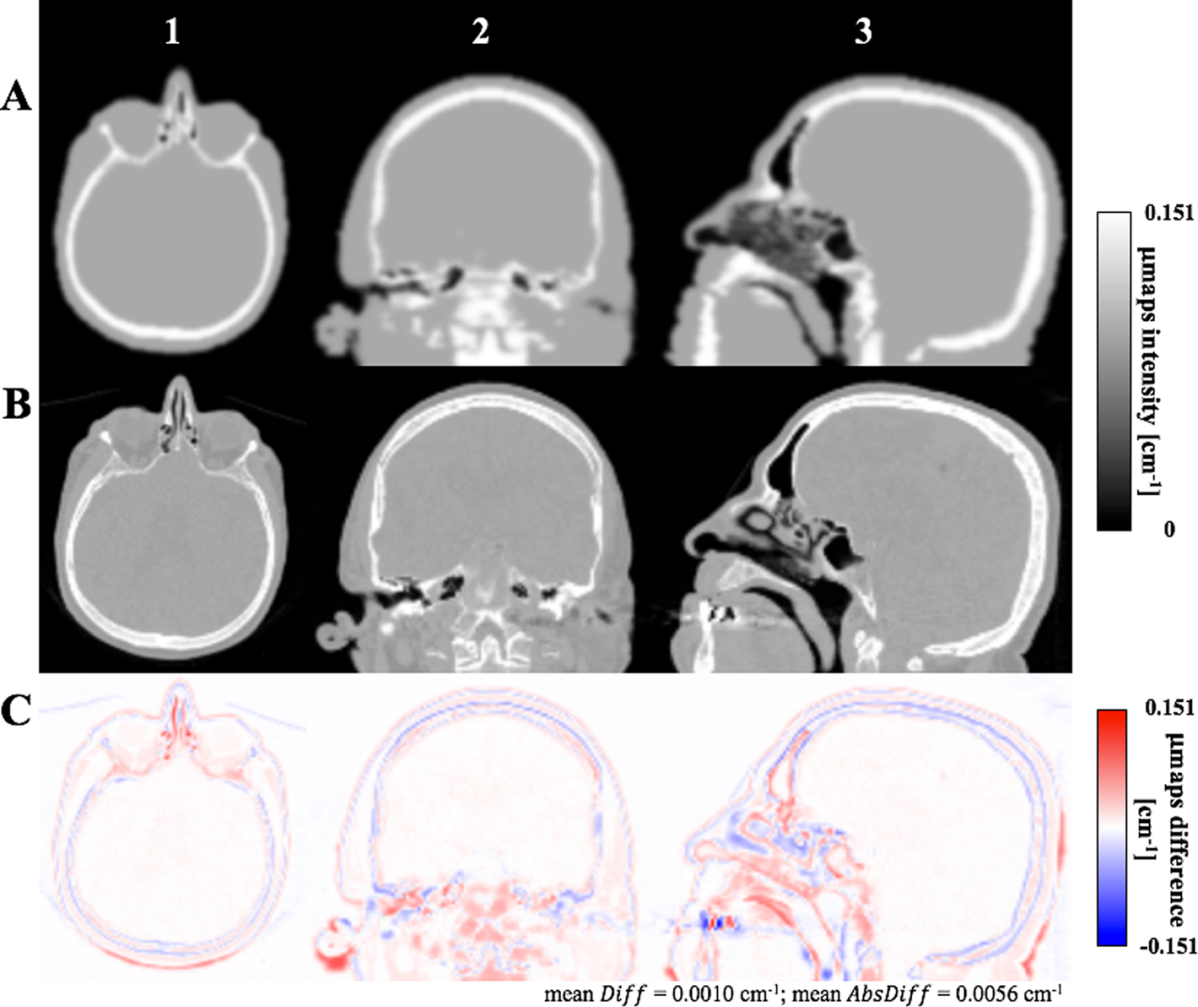
Comparison of attenuation maps from CT group. Comparison of attenuation maps for AC_ref_ (panel **a**) and AC_CT_ (panel **b**) in axial (column 1), sagittal (column 2) and coronal (column 3) view for the subject no. 4 from CT group. Relative error between AC_ref_ and AC_CT_ is depicted in panel **c**

Comparison of AC_ref_ and AC_CT_ maps are shown in Fig. 3. Visual comparison shows very good agreement between the AC_ref_ and AC_CT_. Co-localization of bone and air within the brain is excellent. The differences are seen in skull bones thickness, where AC_CT_ has tendency to overestimate bones, and in sinuses, where the CT and MR co-registration shows large error.

Quantitative comparison between the attenuation maps across all subjects within the CT group is shown in Tables 3 and 4. Table 3 shows the DICE, FP and FN rates within the three tissue classes, i.e. air, soft tissue and bones. Averaged DICE is also calculated. Excellent agreement for air and soft tissue are shown and high similarity for bones between the two maps. The mean FP rate for bones (FP = 0.219) is relatively high, suggesting overestimation of bones in AC_CT_ and this is confirmed by visual inspection in the skull region. Averaged DICE show very good consistency between both methods with the mean value equal 0.917. Table 4 reveals very low mean differences across all subjects between the compared techniques resulted in 0.0004 cm^− 1^ and 0.0064 cm^− 1^ values in relative difference and absolute difference, respectively.

**Table 3:** Different accuracy matrices for the CT-based segmentation method within the MNI space mask

Subject no.	Air			Soft Tissue			Bones			Averaged DICE
	DICE	FP	FN	DICE	FP	FN	DICE	FP	FN	
01	0.974	0.033	0.017	0.933	0.055	0.060	0.792	0.226	0.019	0.900
02	0.991	0.010	0.008	0.962	0.028	0.039	0.831	0.211	0.010	0.928
03	0.983	0.024	0.009	0.955	0.030	0.052	0.837	0.203	0.010	0.925
04	0.988	0.014	0.008	0.956	0.034	0.047	0.797	0.237	0.014	0.914
Mean	0.984	0.020	0.010	0.952	0.037	0.049	0.815	0.219	0.014	0.917
Std	0.007	0.010	0.004	0.012	0.012	0.009	0.023	0.015	0.004	0.013

(Air threshold = − 500 HU; bone threshold = 300 HU for CT images)

**Table 4:** Mean difference (*Diff*) and mean absolute difference (*AbsDiff*) between manual segmentation based attenuation maps and CT based attenuation maps for each subject from CT group

Subject no.	*Diff* [cm^−1^]		*AbsDiff* [cm^− 1^]	
	Mean	SD	Mean	SD
01	0.0013	0.0145	0.0072	0.0126
02	−0.0004	0.0110	0.0059	0.0093
03	−0.0002	0.0127	0.0068	0.0108
04	0.0010	0.0113	0.0056	0.0098
Mean	0.0004		0.0064	

### Comparisons of classification accuracy in air and bone in the PET group

**Fig. 4:**
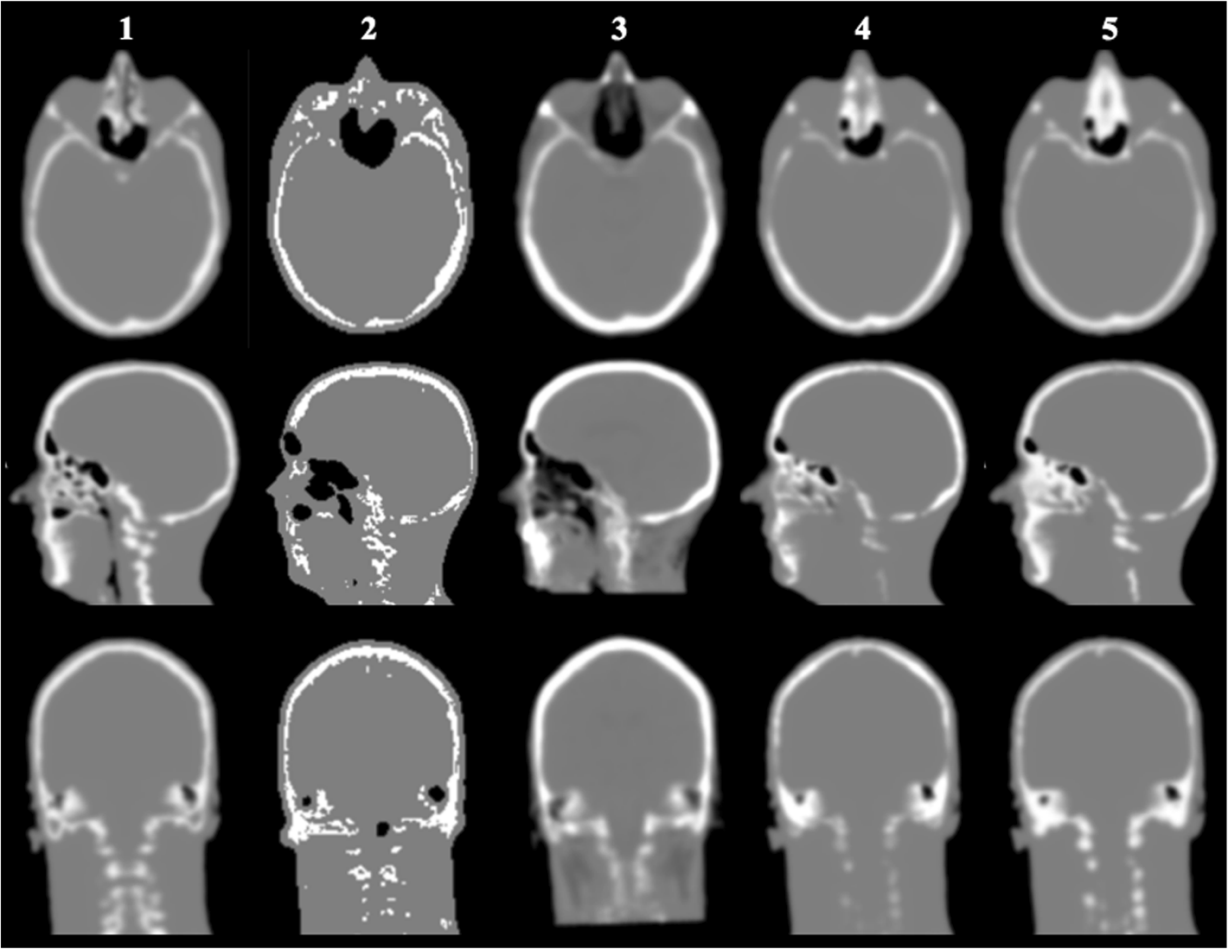
Comparison of attenuation correction maps for the Test participant. Comparison of attenuation correction maps for AC_ref_ (column 1), AC_UTE_ (column 2), AC_UCL_ (column 3), AC_sUTEcont_ (column 4) and AC_sUTEfix_ (column 5) in axial (top), sagittal (centre) and coronal (bottom) view for the Test participant

The attenuation correction maps for the different methods for the Test Participant are shown in Fig. 4. The proposed sUTE method shows improved classification of nasal cavities, temporal bones, and regions around the teeth, when compared with the conventional AC_UTE_ method. The cortical bones are more accurately classified with the proposed method, compared to AC_UCL_ which shows an overestimation of cortical bones. Bones in nasal cavities are slightly overestimated in the proposed method, whereas both the AC_UCL_ and AC_UTE_ methods underestimate bones, but overestimate the size of air cavity in this region.

In Table 5, the proposed method shows excellent accuracy in segmenting air and tissue with a DICE coefficient of 0.985. There were very low numbers of false positive mis-segmented air voxels in the top of the head (FP = 0.007) compared to the other methods. The overestimation of the air tissue shows poorer FN coefficients (FN = 0.022) for the proposed method compared to the AC_UCL_ method (FN = 0.018). The bone segmentation results for the AC_UCL_ and the proposed method show improvements compared to the AC_UTE_ technique. Similar results are observed for false-positive and false-negative analyses across the evaluated methods, with the vendor’s method showing the best FP result and the AC_UCL_ method showing the best FN result. The proposed method, and the AC_UCL,_ method, show better segmentation compared to the AC_UTE_ technique.

**Table 5:** Different accuracy matrices for the different segmentation methods for the PET group

Method	Air			Bones		
	DICE	FP	FN	DICE	FP	FN
UTE	0.953(006)	0.091(015)	**0.006(003)**	0.641(042)	**0.204(028)**	0.431(043)
sUTE	**0.985(002)**	**0.007(007)**	0.022(009)	0.737(017)	0.215(084)	0.277(098)
UCL	0.971(024)	0.041(062)	0.018(011)	**0.780(024)**	0.371(034)	**0.123(023)**

The best results are shown in bold

### Comparison of PET images

**Fig. 5:**
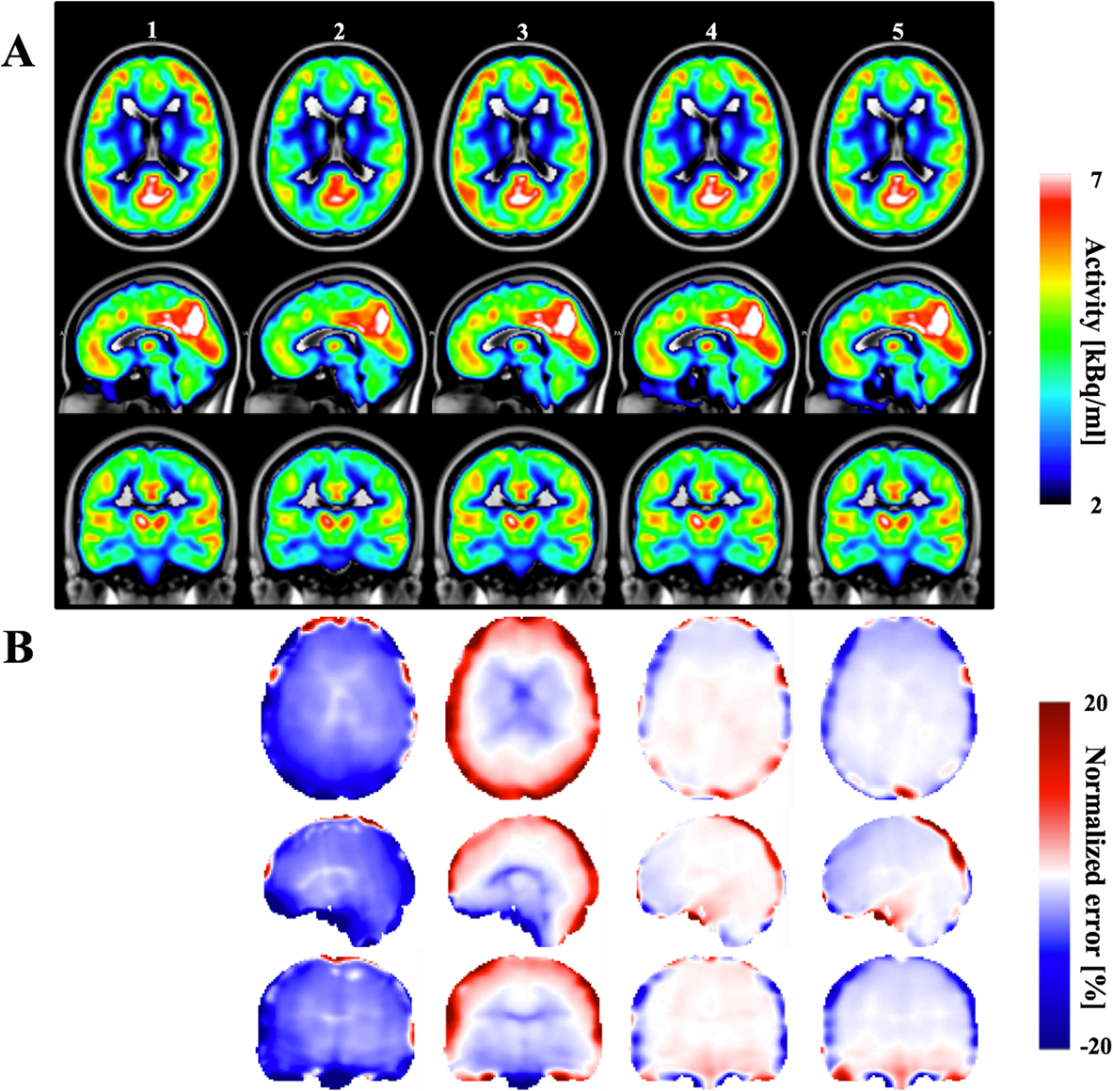
Comparison of PET images for the Test participant. Comparison of PET images for the Test participant (panel **a**) reconstructed with five different attenuation maps: PET_ref_ (column 1), PET_UTE_ (column 2), PET_UCL_ (column 3), PET_sUTEcont_ (column 4) and PET_sUTEfix_ (column 5). Comparison of corresponding normalized error (panel **b**) for the reconstructed PET data is depicted

The reconstructed PET images of the Test Participant using different attenuation correction maps, and the normalized error maps, are shown in Fig. 5. Comparing the reconstructed PET images, the proposed PET_sUTEfix_ and PET_sUTEcont_ show the overall most accurate reconstruction with the lowest normalized reconstruction error. The PET_UTE_ images show a significant underestimation of activity in the whole brain. On the other hand, the PET_UCL_ method shows an increased uptake at tissues adjacent to the cortical bones, especially in the parietal lobe grey matter, up to 20%, and in the brain stem, lateral ventricle and thalamus, where underestimated activity uptake is observed. The proposed methods show reconstruction errors around the cortex due to errors in the cortical bone estimation in the corresponding AC maps. Compared between the two proposed methods, the PET_sUTEcont_ shows overall lower error due to a more accurate bone attenuation coefficient from the R2*-based approaches.

### Comparison of group PET images

**Fig. 6:**
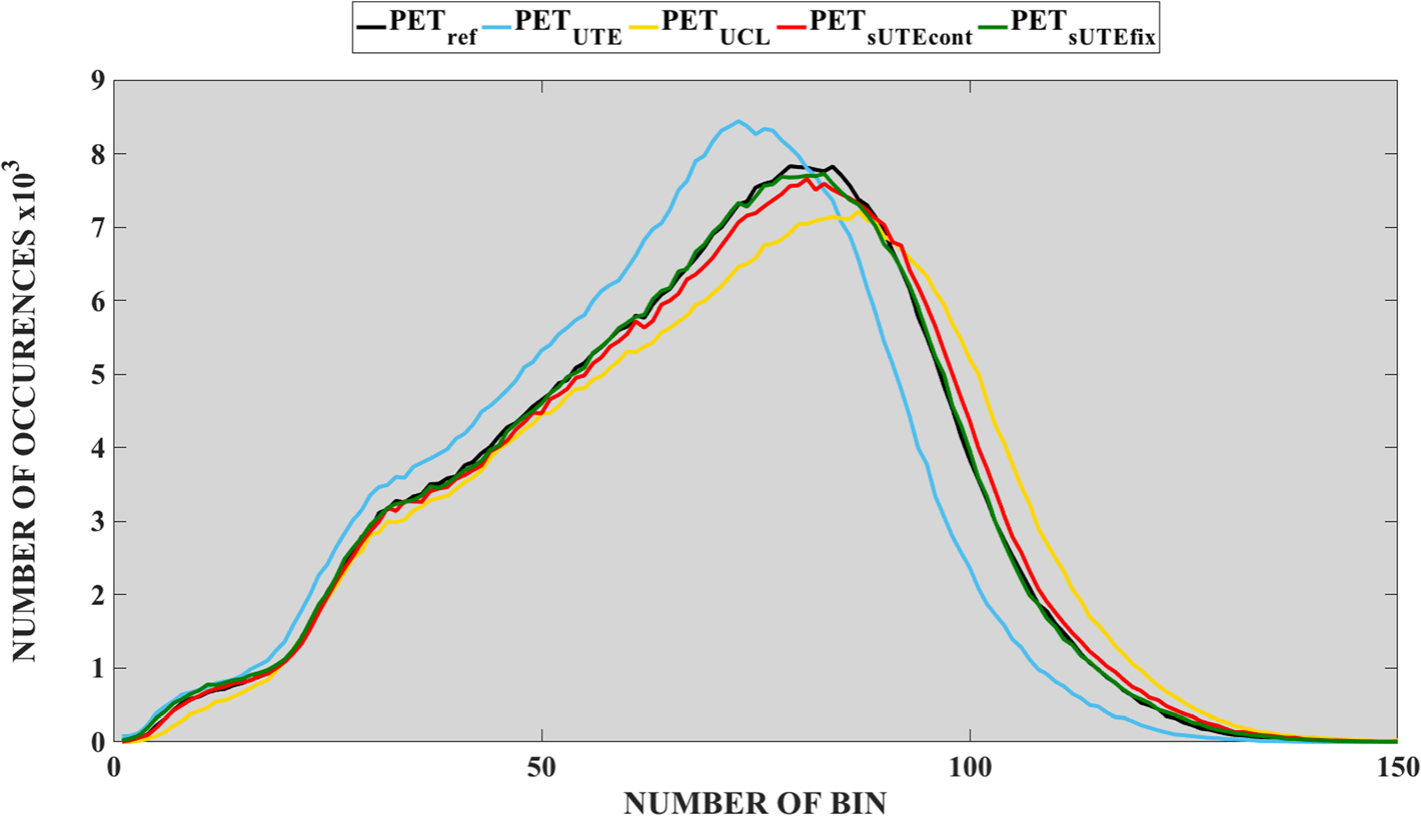
Histograms of the reconstructed PET images. Histograms of the reconstructed PET images within the brain for the group using different methods

Figure 6 shows the histograms of PET images for all participants in the cohort. The PET_UTE_ shows a lower mean uptake compared with other methods. The PET_UCL_ histogram shows a wider spread of uptake values in comparison with the other methods. Compared with the reference PET_ref_ images, both the PET_sUTEfix_ and PET_sUTEcont_ show similar intensity distributions, with the PET_sUTEfix_ image demonstrating slightly more accurate results compared to the PET_sUTEcont_ image.

### Comparison of PET performance in brain regions

**Fig. 7:**
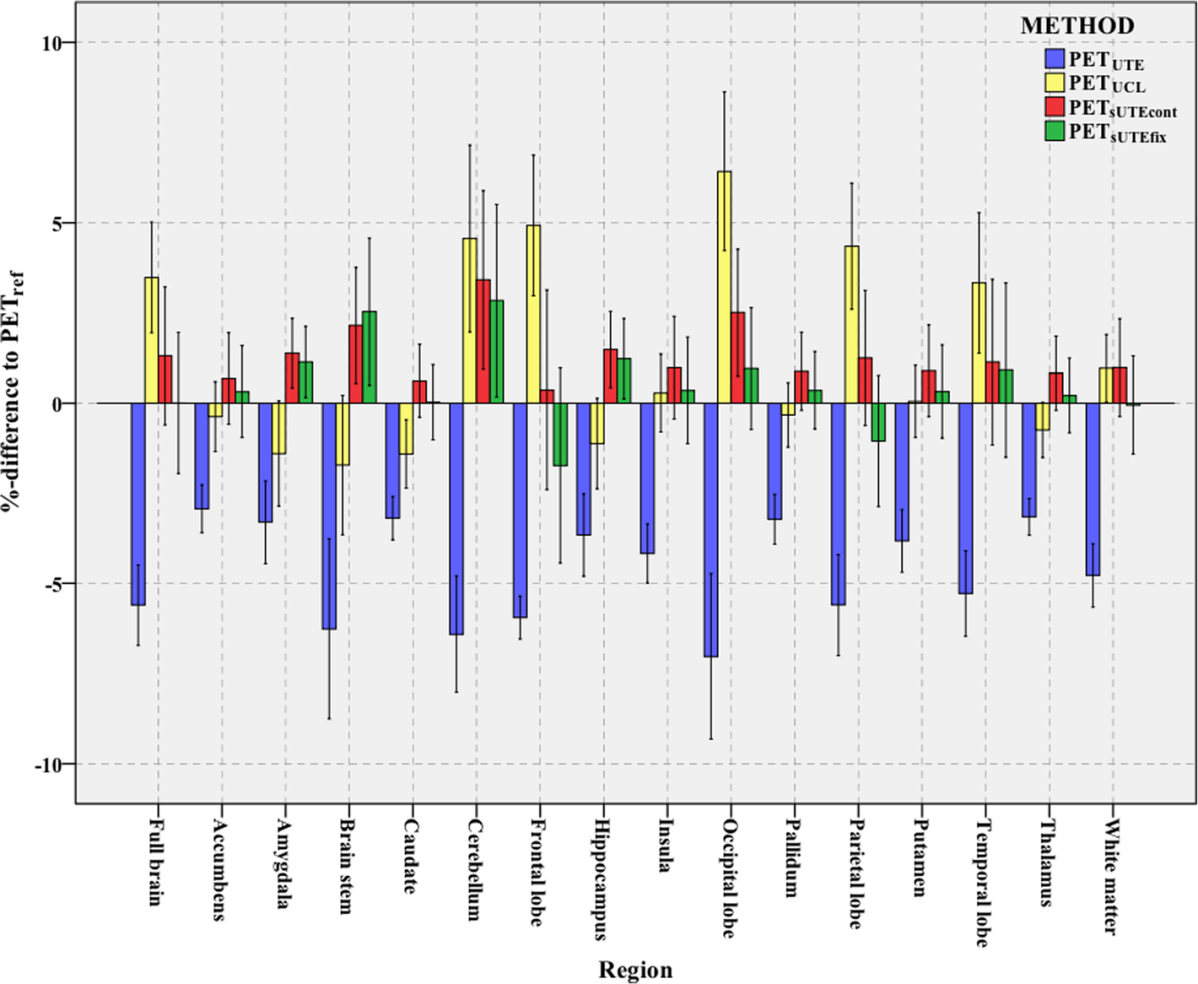
Comparison of quantitative PET measurements. Comparison of quantitative PET measurements in various brain regions with respect to the reference (%-difference ± SD)

The mean *RE%* of the whole brain, and across the brain regions, is shown in Fig. 7. The PET images reconstructed with the proposed attenuation correction method show excellent agreement with the reference PET_ref_ images. The most significant differences are observed in the cerebellum, where the PET_UCL_, PET_sUTEcont_ and PET_sUTEfix_ images overestimate the PET signal by 4.6, 3.4 and 2.8%, respectively, whereas the PET_UTE_ image gives a significantly lower uptake (− 6.4%). The PET_UCL_ method generally overestimates the activity for all brain regions, with a mean of 3.8% for the whole brain. Both the PET_sUTEcont_ and PET_sUTEfix_ images have very similar full brain activity uptake across the subjects compared with the reference. Detailed results are presented in Table 6.

**Table 6:** ROI analysis for the PET group

Region	Method							
	PET_UTE_ - PET_ref_		PET_UCL_ - PET_ref_		PET_sUTEcont_ - PET_ref_		PET_sUTEfix_ - PET_ref_	
	Mean	SD	Mean	SD	Mean	SD	Mean	SD
Full brain	−5.6*	1.1	3.8*	1.5	1.3	1.9	0.0	2.0
Accumbens	−2.93*	0.67	−0.37	0.97	0.7	1.3	0.3	1.3
Amygdala	−3.3*	1.2	−1.4	1.5	1.38*	0.97	1.14*	0.98
Brain stem	−6.3*	2.5	−1.7*	1.9	2.2*	1.6	2.5*	2.0
Caudate	−3.20*	0.60	−1.41	0.95	0.6	1.0	0.0	1.0
Cerebellum	−6.4*	1.6	4.6*	2.6	3.4*	2.5	2.8	2.7
Frontal lobe	−5.95*	0.59	4.9*	2.0	0.4	2.8	−1.7	2.7
Hippocampus	−3.7*	1.2	−1.1*	1.3	1.5*	1.1	1.2*	1.1
Insula	−4.17*	0.82	0.3	1.1	1.0	1.4	0.4	1.5
Occipital lobe	−7.0*	2.3	6.4*	2.2	2.5*	1.8	1.0	1.7
Pallidum	−3.22*	0.69	−0.33	0.89	0.9	1.1	0.4	1.1
Parietal lobe	−5.6*	1.4	4.4*	1.8	1.3	1.9	−1.1	1.8
Putamen	−3.82*	0.87	0.1	1.0	0.9	1.3	0.3	1.3
Temporal lobe	−5.3*	1.2	3.33	2.0	1.1	2.3	0.9	2.4
Thalamus	−3.16*	0.51	−0.74	0.77	0.8	1.0	0.2	1.0
White matter	−4.78*	0.88	0.96	0.94	1.0	1.4	−0.0	1.4

^*^- differences between tested method and PET_ref_ considered statistically significant (*p* < 0.05)

**Fig. 8:**
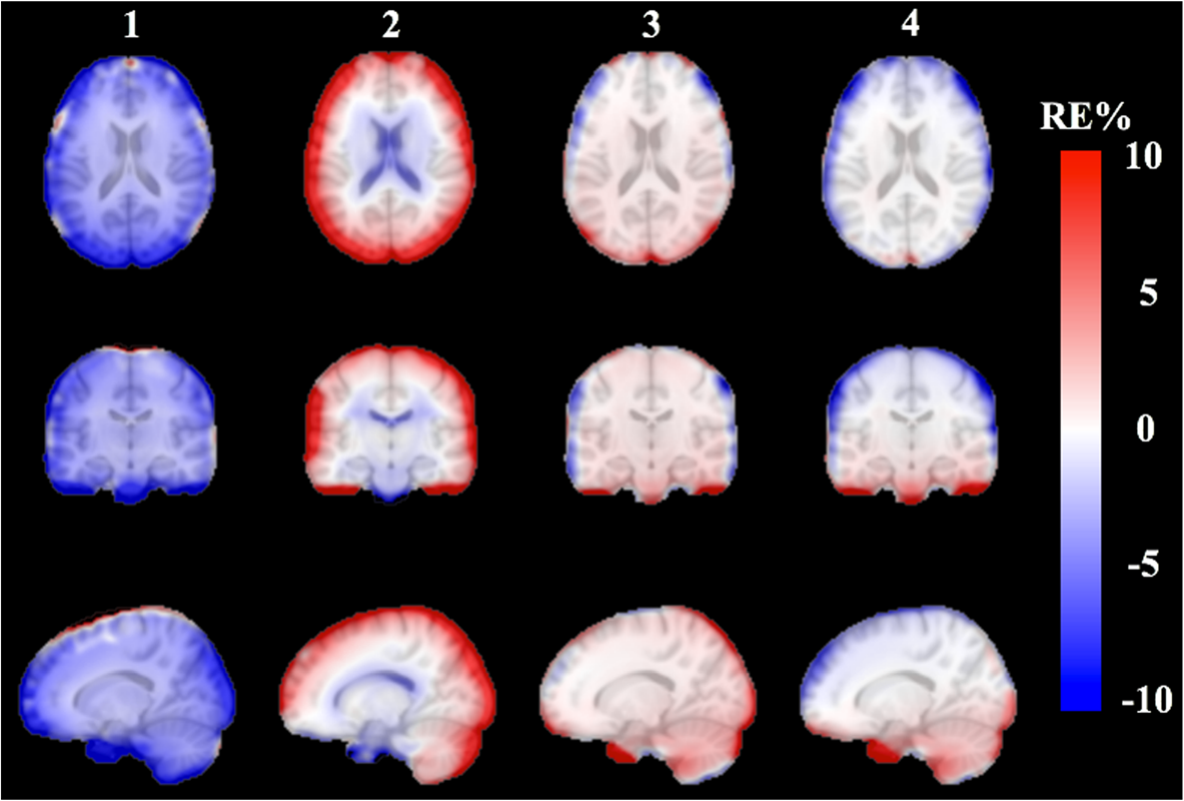
Group averaged *RE%* images in the MNI template space. Group averaged *RE%* images in the MNI template space across all subjects for each method: (1) PET_UTE_, (2) PET_UCL_, (3) PET_sUTEcont_ and (4) PET_sUTEfix_

**Fig. 9:**
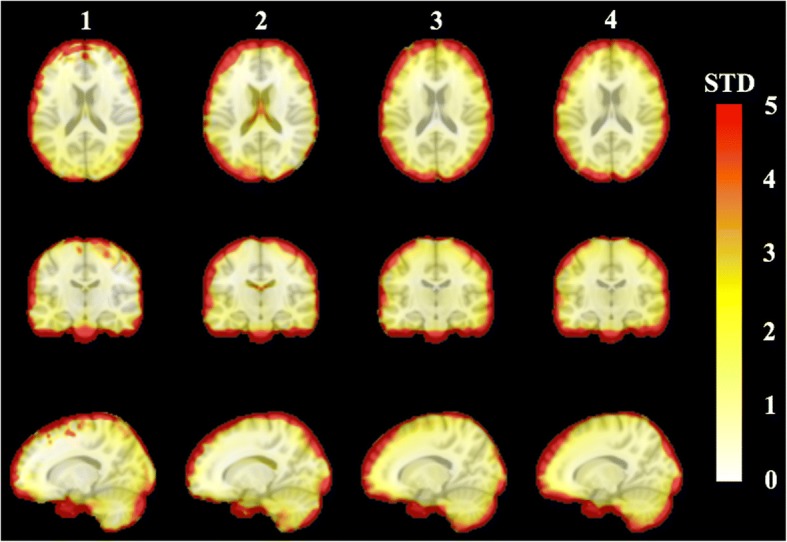
Group standard deviation images in the MNI template space. Group standard deviation images in the MNI template space across all subjects for each method: (1) PET_UTE_, (2) PET_UCL_, (3) PET_sUTEcont_, and (4) PET_sUTEfix_

Compared to the reference PET_ref_, an averaged *RE%* image across all subjects is obtained, as in Fig. 8, and the standard deviation image is shown in Fig. 9. The group *RE%* image (Fig. 8) confirms the superiority of both the PET_sUTEfix_ and PET_sUTEcont_ methods.

### Evaluation of multi-compartment soft tissue AC map

**Fig. 10:**
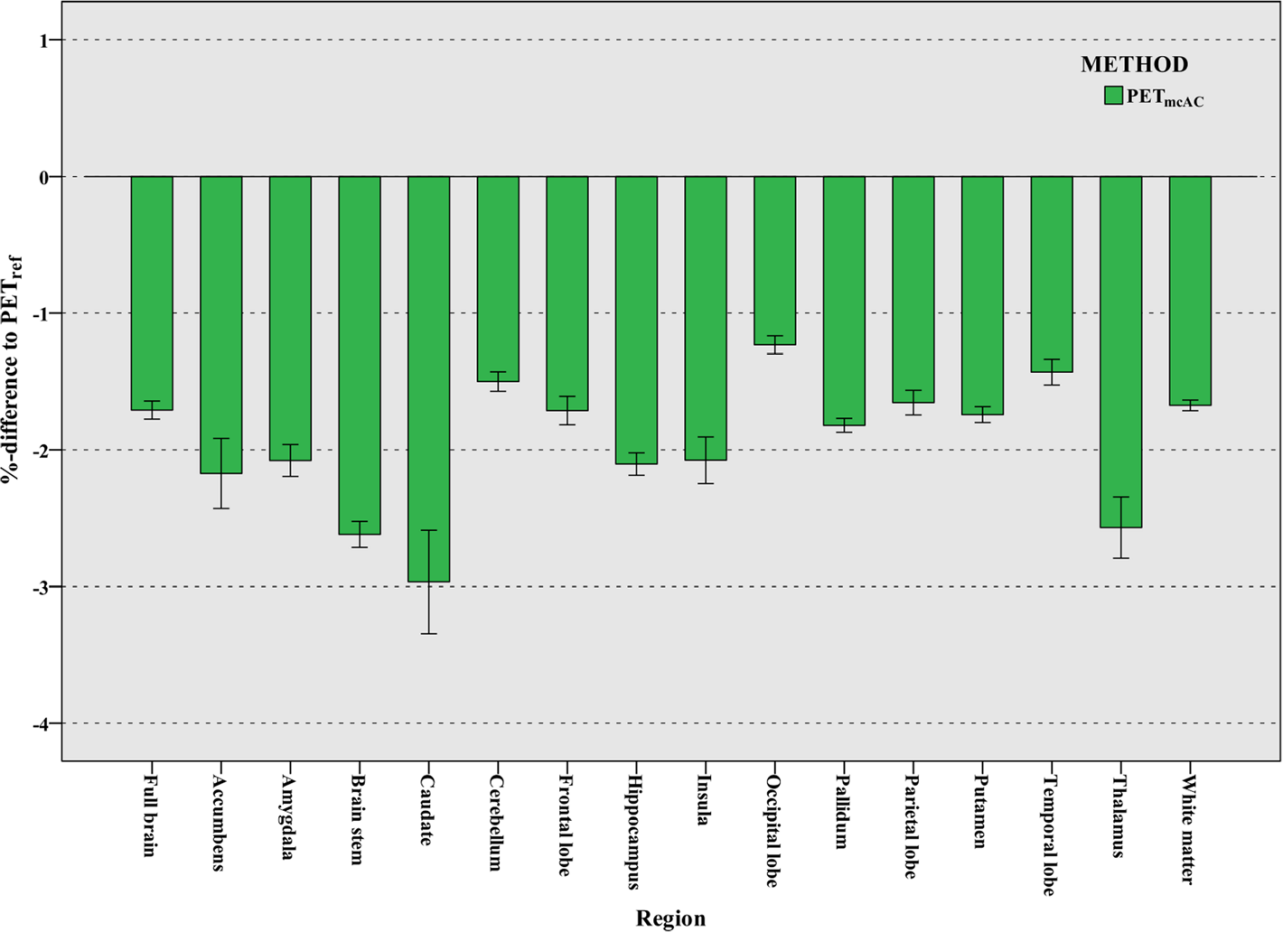
Comparison of reconstructed images. Comparison of reconstructed images using 3-compartment soft tissue AC (PET_mcAC_) and 1-compartment soft tissue AC (PET_ref_)

**Fig. 11:**
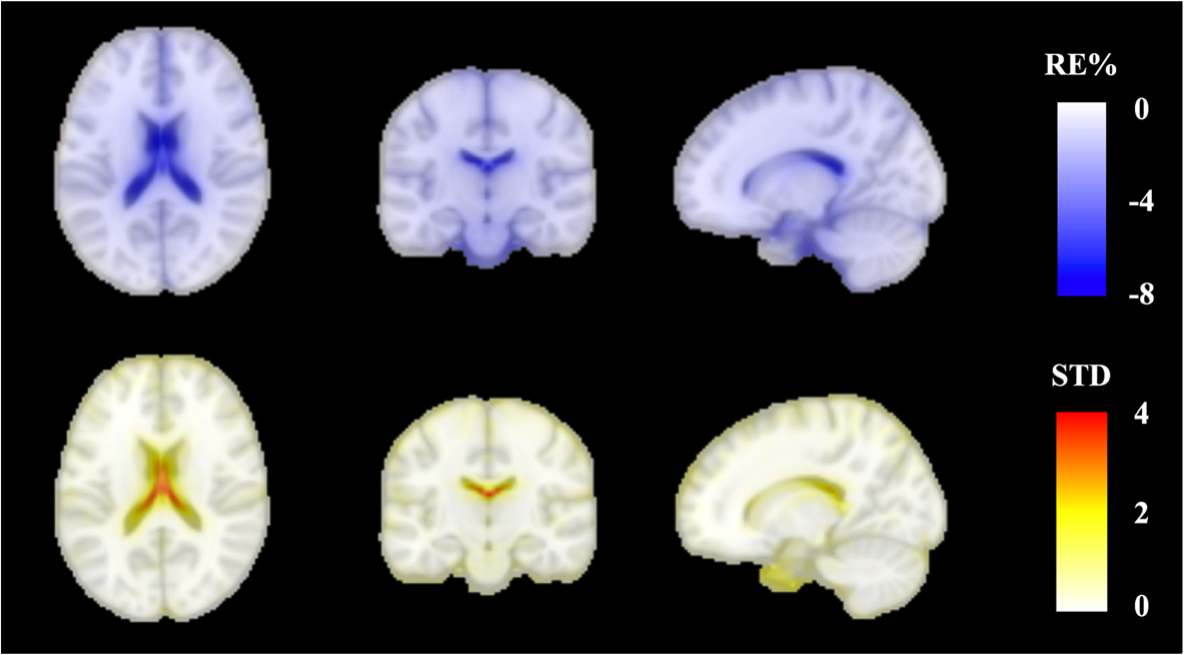
Group averaged *RE%* and standard deviation images in the MNI space. Group averaged *RE%* (top row) and standard deviation (bottom row) images in the MNI space across all subjects for the 3-compartment soft tissue model with respect to 1-compartment soft tissue model

To investigate the impact of multi-compartment AC coefficients in soft tissue, PET_mcAC_ is reconstructed using multi-compartment soft tissue models. The mean *RE%* between the PET_ref_ and PET_mcAC_ images across several anatomical regions and the whole brain is shown in Fig. 10. The reduction of activity estimation is between 1 and 3% for a number of brain regions. The most significant differences are seen in the regions near the ventricles: caudate, hippocampus, thalamus, etc. The average *RE%* and standard deviation images are shown in Fig. 11.

## Discussion

This paper introduces a new PET AC map estimation method for simultaneous PET/MR imaging. Unlike conventional MR-CT atlas based methods, e.g. AC_UCL_, the new method is fully based on MR data. We have created a bone/air/soft tissue template based on the manual segmentation of UTE images using data from five participants. Gaussian mixture models are then used to fit UTE images to generate new AC maps. We show that manual segmentation based attenuation correction maps are comparable to the CT based attenuation maps. Compared with the reference AC map, the proposed sUTE method shows very good results for the whole brain region with the DICE coefficient equal to 0.985 and 0.737 for air and bone, respectively. The proposed method shows significant improvements in reduction of the reconstruction errors, compared with the AC_UTE_ and AC_UCL_ methods.

The proposed AC_sUTEfix_ and AC_sUTEcont_ maps show excellent agreement with the reference AC map (Fig. 4), especially in the region of the cortical bones, where the AC_UCL_ shows an overestimation of the AC coefficients. These differences in AC maps are also confirmed by the false positive and false negative scores in Table 5. Differences in the DICE coefficients, false-negative and false-positive scores, demonstrate the difficulties with bone and air segmentation. In terms of the classification of air cavities in the head and neck, the best results are obtained using the proposed method, albeit with a slight underestimation in the oesophagus. Very low false negative scores for the AC_UTE_ method in the surrounding air are likely to result from significant underestimation of air cavities. The improved classification of air in the new method is due to the excellent air/soft-tissue contrast in the MR UTE template, compared with that in the CT template.

Overall and regional PET performance analyses demonstrate good agreement of the PET_sUTEfix_ and PET_sUTEcont_ images with the reference image. The histograms of PET_ref_ and PET_sUTEfix_ in the group study are the most similar. In the averaged *RE%* images, the overall higher uptake in grey matter adjacent to cortical bones in PET_UCL_ is caused by bone region overestimation and atlas-based attenuation coefficient values which are greater than those used in other methods (particularly in the occipital cortex). Furthermore, the cerebellum is a critical region for proper PET quantitative assessment and kinetic modeling, as it is used as a reference region. The PET_sUTEcont_ and PET_sUTEfix_ images underestimate regions in the frontal pole and frontal gyrus, and overestimate the signal in the postcentral gyrus and the brain stem. All of the methods show varying levels of error compared to the PET_ref_ image in the above-mentioned areas, due to difficulties of accurate classification in the nasal cavities, the base of the skull and occipital bone, which are adjacent to important areas for ^18^F-FDG PET quantification.

We compared the group variability for the three different methods, with all methods demonstrating some variability in the cortical areas, compared to the reference image. This indicates the importance of accurate bone segmentation and the necessity of subject-specific linear attenuation coefficients, especially considering that cortical bone density can vary systematically between individuals. Towards this end, the UTE R2* based method is potentially more accurate, as it measures subject-specific information, whereas atlas-based methods are limited by the richness of their atlas and tend to regress every subject towards the mean.

The selection of a reference AC map for the PET/MR is not straightforward. Due to absence of gold standard transmission scans, the CT based attenuation maps are widely used as a silver standard for that purpose. However, very early study on the PET attenuation correction [57] showed statistically significant differences between transmission based and CT based attenuation maps and resulted in the overestimation of the PET uptake results using CT attenuation maps. As a one of the major issues, Nakomoto et al. indicated the problem of the conversion from HU to 511 keV attenuation correction factors. Furthermore, registration errors between CT and MR can potentially be a source of error in PET/MR attenuation correction as discussed in [53]. In this paper, we have evaluated an alternative way of creating reference AC maps based on anatomical delineation of tissue classes.

Due to the lack of knowledge of the true attenuation coefficients, we have investigated the impact of the number of compartments used for soft tissue attenuation correction. The overall difference is around 2%, compared with the 1- and 3-compartment brain tissue models, where the CSF and brainstem show the greatest differences due to the lower CSF attenuation factor of 0.096 cm^− 1^ in the 3-compartment model.

### Limitations and future work

There are several limitations to this study. The first limitation is the absence of an absolute reference for the attenuation coefficient maps to compare results from different methods. The absolute ground truth reference for PET attenuation correction studies is measured using a 511 keV transmission scan in the PET acquisition [57]. Other studies have used CT scans to measure and compute a reference AC map for 511 keV. However, standard CT images correspond to linear attenuation coefficients for photons with an energy range between 80 and 140 keV, with the extrapolation from the CT to PET attenuation coefficient values potentially introducing a systematic bias. The problem with having an absolute reference might be addressed by TOF MLAA methods which could generate attenuation correction maps estimated from the PET-scanner emission data.

In this study, the reference AC map is generated using manual segmentation of brain tissue, bones and air cavities according to the anatomical landmarks used in standard clinical practice. Similar to other template based methods, there may be potential segmentation and registration errors. However, compared with the templates created from other modalities, the sUTE methods are potentially more accurate to the excellent soft tissue contrast from MRI.

Furthermore, this study includes a small number of healthy participants and focuses on a particular age group. In future work, we plan to investigate the effects of age differences (i.e. bone density differences with ageing) and investigate the attenuation coefficient differences using both PET/CT and PET/MR scanning in healthy and disease groups.

## Conclusion

We propose a novel attenuation correction method for PET data reconstruction, with a common segmentation step, but different approaches for assignment of the bone attenuation coefficients. The first method uses a fixed attenuation coefficient for bone, whereas the second method employs R2* map conversion to determine the PET attenuation coefficients. Visual and quantitative analyses show significant improvements in the quantitative accuracy of the reconstructed PET images compared to standard methods, and improved results compared to other state-of-the-art AC methods for simultaneous PET/MR scanners. Accurate air segmentation is a significant advantage of the proposed method, and potentially enables the reconstructed PET images to be used for quantitative neurological brain imaging. However, further evaluation is needed with radiopharmaceuticals other than FDG, and in larger cohorts of participants, including those with neurological diseases and focal lesions.

## Abbreviations

AC: Attenuation coefficients maps
CSF: Cerebrospinal fluid
CT: Computed tomography
dUTE: Dual ultrashort echo time sequence
FDG: Fluorodeoxyglucose
FN: False-negative
FOV: Field-of-view
FP: False-positive
GM: Grey matter
HU: Hounsfield Units
MLAA: Maximum likelihood attenuation and activity reconstruction algorithm
MNI: Montreal Neurological Institute
MR: Magnetic resonance
OP-OSEM: Ordinary Poisson - Ordered Subset Expectation Maximization algorithm
PET: Positron Emission Tomography
PET/MR: Positron Emission Tomography and Magnetic Resonance
PSF: Point Spread Function
RE: Relative error
SPM12: Statistical parametric mapping software
sUTE: Segmented UTE
SVD: Singular Value Decomposition
TE1: First echo time
TE2: Second echo time
TOF: Time-of-flight
TPM: Tissue probability maps
TR: Repetition time
UTE1: First echo UTE image
UTE2: Second echo UTE image
WM: White matter
